# High speed color imaging through scattering media with a large field of view

**DOI:** 10.1038/srep32696

**Published:** 2016-09-07

**Authors:** Huichang Zhuang, Hexiang He, Xiangsheng Xie, Jianying Zhou

**Affiliations:** 1 State Key Laboratory of Optoelectronic Materials and Technologies, Sun Yat-sen University, Guangzhou 510275, China; 2 Department of Physics, Guangdong University of Education, Guangzhou 510303, China; 3 Department of Physics, College of Science, Shantou University, Shantou, Guangdong 515063, China

## Abstract

Optical imaging through complex media has many important applications. Although research progresses have been made to recover optical image through various turbid media, the widespread application of the technology is hampered by the recovery speed, requirement on specific illumination, poor image quality and limited field of view. Here we demonstrate that above-mentioned drawbacks can be essentially overcome. The realization of high speed color imaging through turbid media is successfully carried out by taking into account the media memory effect, the point spread function, the exit pupil of the optical system, and the optimized signal to noise ratio. By retrieving selected speckles with enlarged field of view, high quality image is recovered with a responding speed only determined by the frame rates of the image capturing devices. The immediate application of the technique is expected to register static and dynamic imaging under human skin to recover information with a wearable device.

Optical imaging is a prime means of collecting information on macroscopic and microscopic world. Naturally, most objects and their surroundings are optically turbid, giving rise to human “visual disturbances”, ranging from astronomical observations through atmosphere^1^ to microscopic imaging in tissues^2^. The view from human’s eyes (or cameras) through turbid media is in the form of a complex and seemingly random speckle pattern^3^,^4^. This speckle pattern was regarded as an imaging degradation of the hidden object mixed with unwanted artifacts.

Imaging through scattering media has anticipated applications in *in-vivo* imaging into deep tissue, photoablation of angiogenic vessels in tumours, and other biomedical uses. Further development would benefit the medical surgery, e.g., noninvasive surgery, endoscopy, physical therapy. Great successes have been made during the last decade with advances in engineering, electronics, and theoretical scattering physics. Optical coherence tomography^5^,^6^,^7^ can obtain sharp images through semi-transparent medium with a gate technique. Wavefront-shaping method effectively focused light deep into biological tissue with an assisted guidestar^8^. Phase conjugation technique delivered arbitrary images through turbid medium^9^,^10^ after measuring its transmission matrix. Adaptive optics techniques^11^,^12^,^13^ near perfectly corrected low-order aberration of the image with spatial light modulator (SLM). Besides that, a more traditional method used in astronomical observation^14^, speckle correlation, attracted attentions in recent years. By utilizing speckle patterns as feedback signals to optimize the focusing through strongly scattering material^15^,^16^,^17^, hidden fluorescent structures can be imaged by raster scanning^18^,^19^,^20^. A non-invasive imaging technique was invented by exploiting the inherent angular correlations in scattered speckle patterns^21^. This technique was further simplified merely with a single-shot of a camera phone^22^. However, to date, no techniques reported allow real-time full color imaging using diffused light with a large field of view (FOV).

Here, we develop a high-speed, full color image technique through a standard scattering medium using broadband white-light as illumination source. The image of an in-home display that is hidden behind a diffuser is rapidly retrieved with a color camera and a reconstruction algorithm. It is the first time, to the best of our knowledge, to experimentally extend the FOV to approach the memory effect (ME) range and the entrance pupil of the imaging system. Benefited from the large FOV and the exclusion of the useless speckles, the system can restore the image with high quality and high speed. Further full color image and video recovery confirms that this technique is promising for various applications, for example peeking through curtain and looking around corners with natural light illumination.

## Principle

Generally speaking, an imaging system, no matter how complicated its internal components are, may be lumped into a single “black box” and be completely described by specifying only the terminal properties of this black box^23^,^24^. Any point at the object plane generates a nearly identical pattern on the image plane, defined as point spread function (PSF), which represents the properties of the imaging system^25^,^26^. The PSF is shift-invariant within the exit pupil and reduces the object-image relation to a convolution equation, 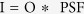, where I and O are intensity distribution (incoherent illumination) on the image and the object planes respectively, 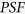 is the point spread function of the imaging system, the symbol * denotes a convolution operation. The FOV of the black box is limited by a limiting aperture, which can be viewed as the image of the most severely limiting aperture when looking from the object space or from the image space through any optical elements. The FOV can be enlarged by positing the limiting aperture in place where the beam diameter is the smallest.

When a scattering medium is introduced into the black box, the PSF becomes a speckle pattern and only shift-invariant within a small angle range^27^,^28^,^29^. The speckle pattern in the imaging plane is the superposition of all PSFs from arbitrary point sources on the object. The imaging process can be denoted as a correlation function of the PSF and the object distribution function^27^,


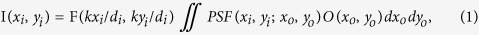


where k is the wavevector, the subscript i and o denote the image and the object plane respectively. 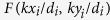 is defined as a form factor function which acts as a field stop placed in the focal plane to limit the observed FOV. Although the scattering medium would possess a large aperture, it still acts as the limiting aperture of the black box because of the F function. The F function is an angular correlation function of the emerging speckle patterns of two incident wavefront 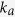 and 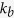,


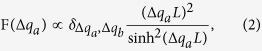


where 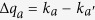, 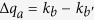, L is the is the effective thickness of the turbid medium, 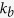 and 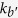 is the corresponding output wavevector. The F function has a bell shape curve of angular correlation, which is defined as the ME range. It means that the information of object has nearly the same speckle distribution in the imaging plane within the ME range. The imaging process through scattering medium is valid only within this range, leading to a small angular FOV.

**Figure 1:**
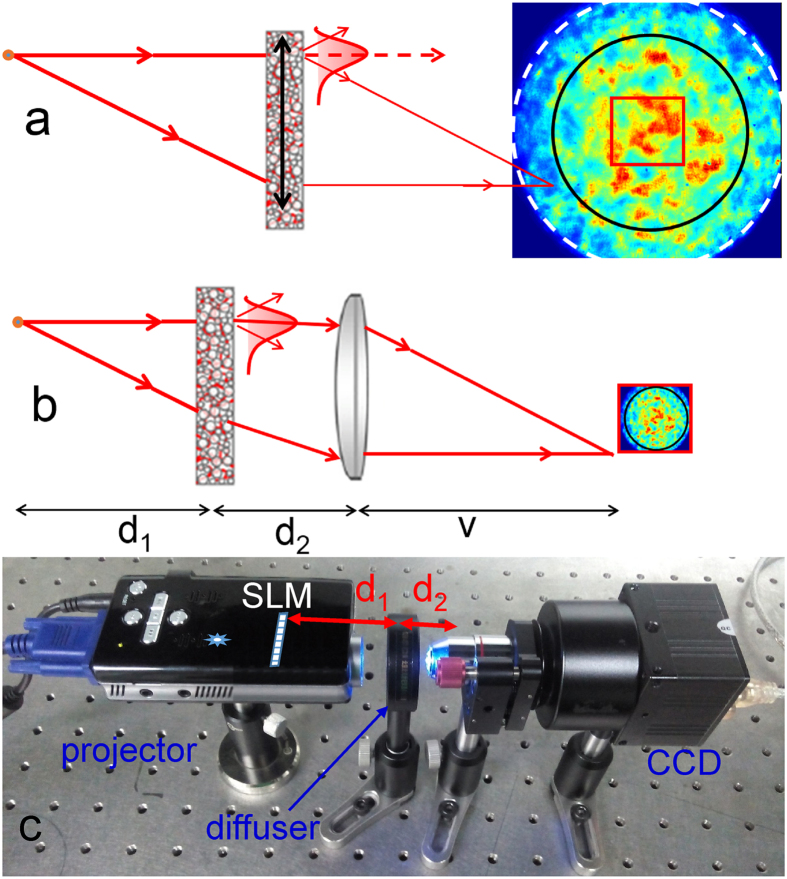
Schematic of the correlation imaging through a scattering medium without (**a**) or with (**b)** the assistant of lenses. White dash circle: Exit pupil; Black circle: View size; Red rectangle: CCD size. (**c)** experimental setup. A white light LED projector (without lens) acts as the hidden object.

The way to enlarge the angular FOV is not to locate the scattering medium to the place with small beam diameter, but with the smallest wavevector difference between the incident and output beams. For conventional speckle correlation imaging, as shown in Fig. 1a, the scattering medium acts as a scattering lens to deflect the wavevector of the incident light to the imaging capture device. In this circumstance, most speckle patterns cannot be used for imaging but result as background noises, which degrades the correlation. Moreover, the speckle pattern from the scattering medium is normally larger than the exit pupil (white dash circle) of the black box and shows up as a halo with a cut-off diaphragm. The ME range viewed from the image space (black circle) should be smaller than the exit pupil, which becomes the most severely limiting aperture of the optical system. However, the CCD camera (red rectangle) is in general placed away from the scattering medium, giving rise to the corresponding field angle smaller than the ME range. Correlation function will be compressed further because the CCD can only capture part of the correlated speckles with limited sensor area. These are the main reasons that the reported angular FOV^11^,^13^ was much smaller than the ME range.

In our previous work, He *et al.* proposed a beam scanning method to enlarge the FOV by tilting the incident illumination light beam^13^ which ensured that the scattering light is mostly concentrated in the ME range and around the central tendency of statistics of speckle intensity. However, additional limitations on the FOV are introduced by the imperfect conjugating placement of the SLM^11^. A new strategy is taken here for a larger FOV equivalently to inserting two lenses, as shown in Fig. 1b. One is used to collect most of the scattering light for imaging and the other to demagnify the image in the entrance pupil to fit the CCD size. This strategy not only enlarges the FOV to approach ME range but also increases the correlation of the speckle pattern within the exit pupil.

## Experiment

An experimental setup for imaging through a scattering medium is presented in Fig. 1c. A LED projector without imaging lens is used as the hidden object, which is equivalent to illuminate a amplitude only SLM or a color slide by a white light LED^30^ (The spectrum of the light source is typical a phosphor-based white light LED whose spectrum covers the visible range.) and to project the diffracted pattern onto a standard scattering medium (Newport 80° circular light shaping diffuser, D = 25.4 mm). As mentioned above, the function of objective lens is to collect most of the scattering light for imaging and the C-mount adaptor with a couple lens is used to demagnify the image to fit the CCD size.

**Figure 2:**
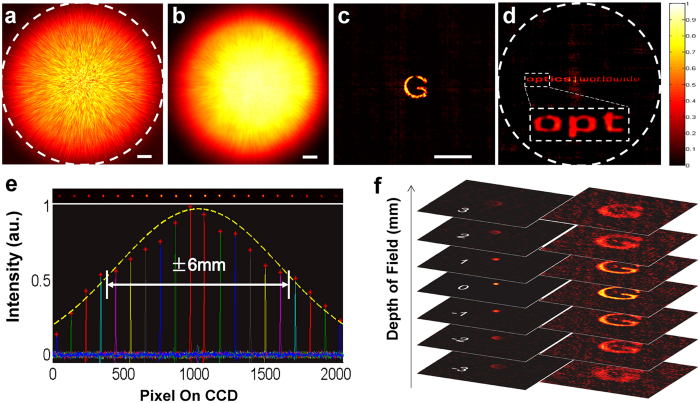
Experimental imaging through a standard scattering medium. (**a**) Reference speckle pattern (PSF) of a single pixel on projector, the white dash circle denotes the exit pupil. (**b**) Speckle pattern of unknown object on projector. (**c**) Retrieved image from a and b by a deconvolution algorithm. (**d**) Large view imaging of a resolution target (signed as ‘optics | worldwide’) to confirm the FOV size. The insert dash rectangle shows the first three letters at the edge of the FOV. (**e**) Measurement of the FOV by shifting a point target along x axis, the measured FOV is ±6.0 mm (±75.0 mrad). **(f**) The defocus-like properties of retrieved images. Scale bars: 200 pixels.

A monochrome CCD is firstly applied to demonstrate the imaging restoration. A rigorous ME measurement^28^ (shown in supplement) confirms that the bell shape curve of angular correlation drops to a half maximum at angle of ±80 mrad @632.8 nm. Before capturing the object speckle pattern, the speckle pattern of a single pixel of the projector or of a 100 um physical pinhole is recorded as PSF of the system (Fig. 2a). The images of the object can then be recovered immediately with a deconvolution algorithm between its scattering patterns (Fig. 2b) and PSF because the F function can be approximated to 1. As shown in Fig. 2a,b, the speckle pattern within the exit pupil, which can be obviously observed as a vignetting, has been zoomed in or out to fit the corresponding ME range. Although the PSF and the object speckle pattern captured successively in Fig. 2a,b are totally blurred, the reconstructed image (Fig. 2c) accurately restoring the setting image (a letter ‘G’) of the projector. The reconstruction image is of high signal noise ratio (SNR) because the speckle pattern of the object is highly correlated with the speckle of the pinhole (PSF) within the ME range.

The FOV of our system is measured by calculating the normalized intensity of the reconstructed image when translating a pinhole along x axis. As shown in Fig. 2e, the intensity of the reconstructed image gradually declines as the pinhole deviates from the original position. When the pinhole is shifted to x = ±6.0 mm, the intensity of its reconstruction image drops down to 0.5. Hence the FOV of this system is ±75.0 mrad (The distance between the object and the diffuser is d_1 _= 80 mm, so the FOV = ±6.0 mm/80 mm = ±75.0 mrad). Owing to the large FOV, the image of a large object (part of 1951 USAF resolution target from Edmund Optics) is reconstructed by a single shot. The result is shown in Fig. 2d, by optimized arrangement of the collection lens, the FOV of the system is just slightly smaller than its exit pupil (white dash circle in Fig. 2d). The first three letters ‘opt’ is enlarged and show a high contrast and high SNR even at the edge of the FOV range. As a consequence, when an imaging system contains a scattering medium, the FOV is double restricted by the ME of the scattering medium and the entrance/exit pupil of the system. The smaller one, in most cases is the ME, results a more severe limiting. In our cases, the FOV angle and ME are ±75.0 mrad and ±80.0 mrad, respectively, while the entrance pupil in the object plane (calculated from the exit pupil in the image plane) is ±112.0 mrad. The FOV should be smaller than the exit pupil, because the deconvolution is distinguishable when two speckle patterns have enough overlapping areas, the center of which separated within the exit pupil range. In the axial direction, the reconstruction is valid when the object is placed near the reference plane within the depth of field, or FOV in Z axis for easy understanding, as shown in Fig. 2f. By moving the object along z axis and reconstructing its image with the same PSF (Fig. 2a), it is interesting to note that the retrieved images have the same defocus-like properties as a common lens. When the object is departing from the reference plane, the recovered imaging fades into a background noise gradually rather than abruptly. This property can be interpreted as the result of speckle correlation for different z position, or “Memory-effect” in z direction^11^.

## Color image and Video

The ultimate goal of any imaging technique is of high resolution, high color fidelity and real-time (or high speed), and this is also the objective of imaging through scattering media. Although the image restoration through turbid media has achieved great success, the optical imaging system was in general too complicated to be adaptable to broadband light illuminating. Any wavelength sensitive device or object, i.e., phase plane, SLM and fluorescence labeling, has limited usable spectral range. There have been only a few reports utilizing multi-wavelength imaging or color imaging through scattering media^31^,^32^. Our technique of imaging restoration does not utilize wavelength sensitive component hence is applicable to broadband illumination and/or detection. For a normal scattering medium without absorption, a colorful imaging can be readily obtainable from the deconvolution process. When the diffuser have a large ME range and enough speckle de-correlation bandwidth, the reconstruction is possible with broadband illumination, which lead to a lower contrast on broadband PSF and lower SNR. The theoretical depiction and experimental demonstration of this feature is presented in Supplementary (Section 3 and 4).

**Figure 3:**
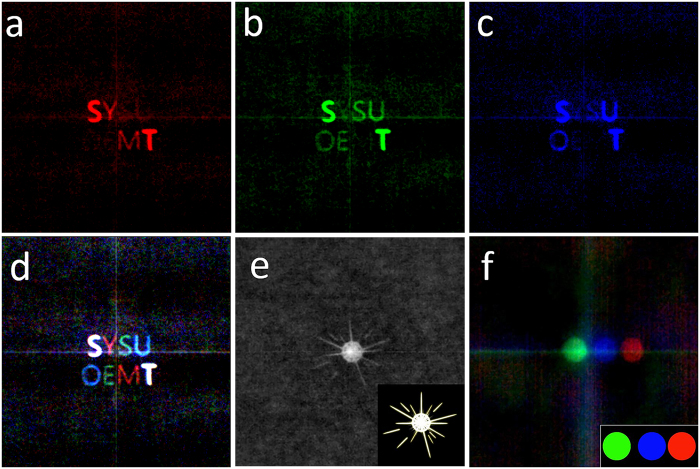
Color images and video restoration. Three primary-color images of **d** on red (**a**) green (**b**) blue (**c**) and a composited color image (**d)**. Restoration of (**e**) a monochrome video (a sparkle cartoon) and (**f**) a color video (consist of three color circles). The inserts in (**e)** and **(f)** are the original videos (See the supplementary movies).

A commercial color CCD, which can separate light into three color channels with built-in color filter, is now mounted on for data acquisition, as shown in Fig. 1c. The speckle patterns of reference point and object are recorded and automatically split into three color channels (R-G-B). The reconstruction procedure for each color channel is the same as the previous monochrome one. Results of three primary-color restoration images is shown in Fig. 3a–c respectively. The three primary-color images composite a full color image (shown in Fig. 3d), including white, red, green and blue elements. There are no chromatic dispersion devices in our imaging system and the CCD is designed for human vision recording. It is promising to cover the whole visible wavelength and to provide a true color, high fidelity imaging function.

For real-time imaging, the reconstruction algorithm is improved (refer to the discussion section). The time assumption for retrieving image from speckle patterns is typically about 1 millisecond, which is negligible comparing to the period of video recording. Hence the system actually works at the video rate. As shown in Fig. 3e,f, a monochrome video and a color video are reconstructed synchronously with the display of the projector. The reconstruction time consumed for the color video is about three times of the monochrome one.

## Discussion

The technique developed in this work is based on the fact that a thin scattering medium can be considered as a shift-invariant system in a narrow range, which is also the basic assumption of most of the autocorrelation method^22^ reported in recent years. So part of the conclusion that derived from the autocorrelation method is applicable here. Although non-invasive single-shot imaging in real-time can be realized with a large FOV, it is only applicable to a narrowband source as required by the nature of correlation measurement. In this case, the spectrum of light source is not a critical factor when the PSFs were obtained with same illumination on the objects. As the ME range of a thin scattering medium is wavelength dependent according to Eq. (2). An additional experiment (see the supplement) shows that a narrower band light source (with a filter (±10@532 nm) would lead to a larger FOV and a higher SNR than the broadband (white LED light) source. But a broadband light source is necessary to provide trio-color channels with high energy efficiency and high fidelity.

The proposed method in this letter needs an off-line construction to get the final imaging result. It has the benefit of digital image preprocessing to improve the quality^21^,^22^, while it is usually time-consuming which is harmful for the application of living tissues observation and real-time surveillance. By parallelization program with a commercial GPU (NVidia GTX 970), the whole process can complete in 1 millisecond. The negligible time consumption makes the system possible for no-delay video reconstruction and real-time recording and display.

**Figure 4:**
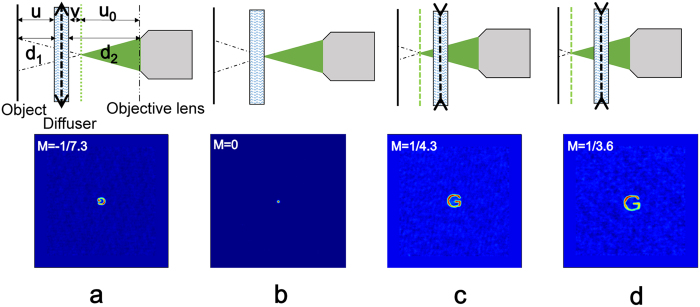
Scattering Lens evolves from a convex lens to concave lens when the distance between the objective lens and the scattering lens is changed.

A scattering medium can be considered as a scattering lens system within the ME range. Its magnification can be calculated by 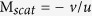. In our case, an optical system is introduced after the scattering medium. So the total magnification of imaging system can be described as 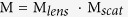
according to geometrical optics, where 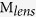 denotes the magnification of the objective lens. It is interesting that the behavior of the whole system will variant according to the distance between them. That is equivalent to say that the focal length of the scattering lens can be changed by adjusting the distance between the objective lens and the scattering medium, as shown in Fig. 4. The focal length of scattering lens depends on the lens equation as 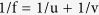. When d_2_ > u_0_ (v > 0), the scattering lens acts as a convex lens with a positive focus length and a negative magnification. An inversed image (Fig. 4a) is obtained on the CCD. The role of scattering lens can be changed to a concave lens when d_2_ < u_0_ (v < 0), as shown in Fig. 4c,d.

## Conclusion

In conclusion, we propose and demonstrate a new strategy to look through scattering medium for full color video. By properly tailoring the speckle image with suitable optical path and detection, the FOV can be extended to approach the ME range and the entrance pupil of the imaging system. The image restoration process with deconvolution algorithm can significantly speed up the imaging recovery, allowing a static and dynamic reconstruction of the image behind the turbid material. Benefited from the large FOV and the exclusion of the useless speckles, the system can restore the image of the hidden object with high quality. This broadband illumination and detection technique gives rise to a capability of *in-vivo* imaging into deep tissue, hence showing immediate application for optical diagnosis with additional spectral fingerprint information^33^,^34^.

## Additional Information

**How to cite this article**: Zhuang, H. *et al.* High speed color imaging through scattering media with a large field of view. *Sci. Rep.* **6**, 32696; doi: 10.1038/srep32696 (2016).

## Supplementary Material

Supplementary Information

Supplementary Video 1

Supplementary Video 2
