# Supplementary material for: High speed color imaging through scattering media with a large field of view
Source: Sci Rep. 2016 Sep 7;6:32696. doi: 10.1038/srep32696 (PMC5013408; doi:10.1038/srep32696)
Supplement: Supplementary Information [file srep32696-s1.pdf]

SUPPLEMENTARY INFORMATION

High speed color imaging through scattering media with a large field of view

Huichang Zhuang[1, †], Hexiang He[2, †], Xiangsheng Xie[1, 3, *], and Jianying Zhou[1]

[1]State Key Laboratory of Optoelectronic Materials and Technologies, Sun Yat-sen University, Guangzhou 510275, China

[2] Department of Physics, Guangdong University of Education, Guangzhou 510303, China

[3]Department of Physics, College of Science, Shantou University, Shantou, Guangdong 515063, China

†These authors contributed equally to this work.

***Corresponding author: xiexsh3@mail.sysu.edu.cn**

1. Verification of Memory-effect (ME)

The sketch of the experiment to verify the ME is shown in Figure S1 (a). The speckle patterns of the scattering medium illuminated by a collimated He-Ne Laser beam are captured by a CCD which located *d* away behind the sample. Figure S1(c) is the speckle pattern when the incident angle of the laser beam is normal. By tilting the incident laser a certain angle of Θ, the speckle pattern shifts accordingly while its distribution almost the same [Figure S1 (d), (e) and (f)]. The correlation coefficient between the original speckle pattern [Figure S1(c)] and the shifted speckle pattern (the shift of speckle should be corrected at first) is calculated as a function of the tilted angle Θ [plotted in Figure S1 (b)]. It is shown that the correlation drops to 1/2 when Θ=80mrad. So the linear shift-invariance can be approximately satisfied in a small range of incident angle, referred to as the ME range. And the measurement was fitted with

$$C(qL) = \left[qL / \sinh(qL) \right]^2, \quad (1)$$

as shown in Figure S1(b) , where $q=2\pi\Theta/\lambda$, Θ is the tilting angle, λ is the wavelength of light, and L is the effective thickness of the turbid medium. The measurement result shows that within the tilting angle of ±80 mrad, the scattering speckle is highly correlated. The fitted L is 1.6 μm.

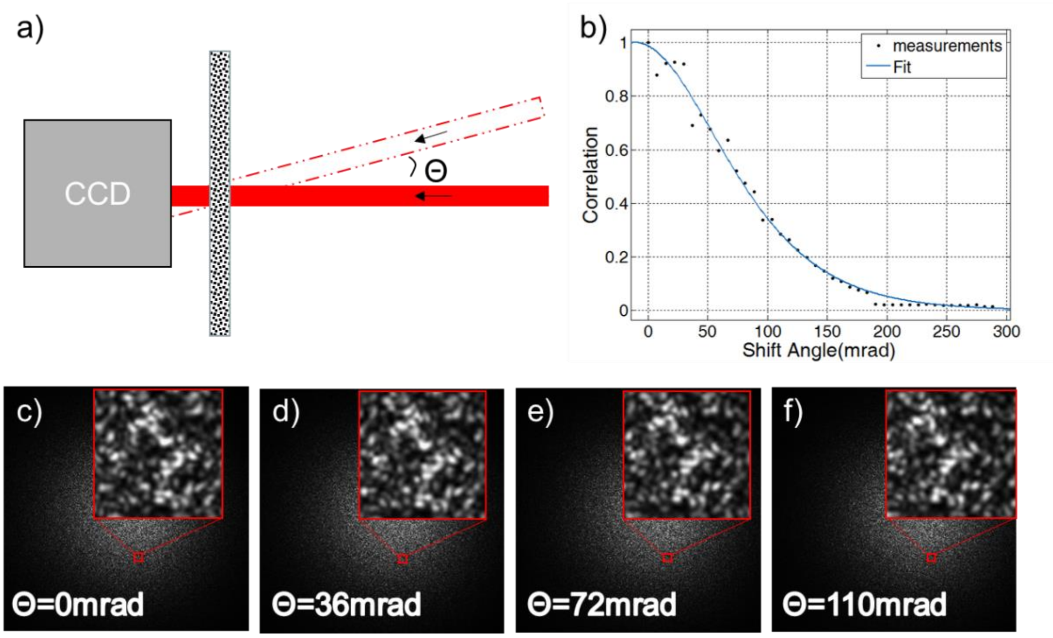

Figure S1. Verification of the optical memory effect of the scattering sample. (a) The schematic diagram of the experiment. (b) The experimental data (solid black dot) and fitted curve (blue line) of the correlation coefficient at different tilted angle Θ. (c) ~ (f) speckle pattern with an incident beam of angle Θ=0, 36, 72 and 110mrad respectively.

2. Brief description on the de-convolution technique with a Wiener filtering algorithms

In a thin scattering system, the speckle pattern generated by placing a narrowband point light source on the object plane can be considered as the point spread function (PSF). According to the optical ME, any other point light source in the vicinity results in a speckle pattern that is highly correlated with the PSF. A thin scattering medium itself can be regarded as a shift-invariant system within the ME range. A more general pattern *O* in the object plane can be considered as a set of object points. The speckle pattern of *O* equals to the convolution of *O* with the PSF. Thus *O* can be reconstructed by a non-blind deconvolution method.

The correlation between the PSF and the speckle pattern is low when the part of object is beyond the ME range, leading to a lot of noise in the reconstructed image. In this situation, several deconvolution algorithms can be adopted to reduce the de-convolution noise. Wiener filtering algorithms is chosen to reconstruct the tested object. It provides acceptable results with a relatively low computation requirement. A typical wiener filtering can be calculated by:

$$\mathrm{F}\ \{\mathrm{O}\} = \frac{\mathrm{F}\ \{\mathrm{PSF}\}^{*}}{\left|\mathrm{F}\ \{\mathrm{PSF}\}\right|^{2} + k} \mathrm{F}\ \{\mathrm{I}\}, \tag{2}$$

where *I* is the speckle pattern of the tested object, *k* is an adjustable parameter of noise. The value of *k* is usually set to 100~1000 in the experiment and the tested object can

be reconstructed by an inverted Fourier transform $U = F^{-1}\{U\}$. One complete computational cycle to reconstruct an image contains 2 Fourier transforms, 1 inverted Fourier transforms and a matrix division, which requires modest computation power. By parallelization program with a commercial GPU (NVidia GTX 970) of a personal computer, the whole process can complete in 1 millisecond.

3. Properties of the diffuser and measurement on the spectral decorrelation bandwidth

The scattering medium used in the experiment is a Newport 80° circular light shaping diffuser (made by Newport Corporation). They have a surface with completely random, non-periodic structures made by holographically replicated on a polycarbonate surface from a holographic mask. Hence its effective thickness is depended on this structural layer and is about 1.6 μm based on the ME measurement, which is in accordance with the result reported in the supplementary of reference[1].

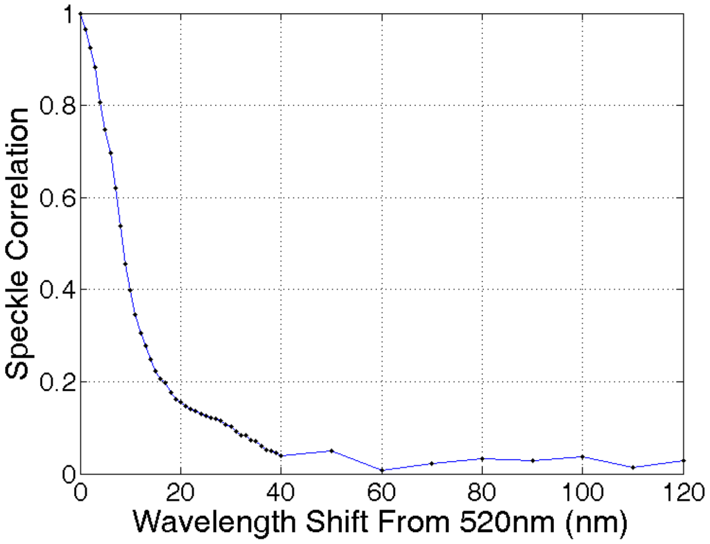

Figure S2. Measurement of the spectral speckle decorrelation bandwidth.

By changing the wavelength length of the incident light source, the speckle patterns of the diffuser will vary and the correlation coefficient with the original one will drop. The Full-Wave-Half-Maximum of the curve of correlation at different wavelengths is defined as the speckle decorrelation bandwidth (SDB). The SDB is the prerequisite to the scientific consideration when broadband light source was applied. The SDB is measured by a similar experimental setup as Figure S1 (a). The beam width is controlled by putting a pinhole in front of sample. A beam from a Halogen-wolfram light passing through a monochromator is used as a tunable light source with the bandwidth smaller than 2nm. By tuning the tested wavelength from 530 nm to 650 nm, the correlation coefficient between the present speckle pattern and the reference speckle pattern at 530 nm is plotted in Figure S2. The measured SDB is 10 nm. Since the diffuser can be consider as a single scattering layer, the mean free path of the sample is untested. However, our sample is not specialized. Its SDB and

effective thickness (L in Eq. (S1)) are comparable to a 0.5 mm thick chicken breast muscle[2,3] (SDB=20nm, L=7μm). The application of the proposed method is mainly restricted by the ME range of the scattering medium. Thus any other scattering samples with similar scattering properties as the one used in our experiment are supposed applicable, such as a rough surface, thin tissue layers, ground glass, thin mist and so on.

4. Discussions on the situation of broadband illumination

4.1. Basic principle of de-convolution image reconstruction through a thin scattering medium under broadband illumination.

Within the linear correlated range of the scattering medium, the speckle pattern of an object O equals to the convolution of O with the PSF. That is

$$I = O * PSF, \tag{3}$$

where I is the speckle pattern on the image plane. This result is restrict to a system with an incoherent monochromatic light illumination. If the object is illuminated by a broadband light source, Eq. (3) is satisfied only for every specify wavelength λ. Here two types of recording devices are discussed. Firstly, for a monochrome camera, it cannot tell apart the information of different wavelength. The final speckle pattern I_{mono} appears as the superposition of speckle patterns at different wavelength

$$I_{mono} = \sum_{\lambda} I(\lambda) = \sum_{\lambda} \left[O(\lambda) * PSF(\lambda) \right]. \tag{4}$$

For a gray scale object $O(\lambda)$, it is considered that all wavelength channels show a same spatial distribution. So that Eq. (4) turn out to be $I_{mono} = O * \sum_{\lambda} PSF(\lambda)$. Here, $\sum_{\lambda} PSF(\lambda)$ is the speckle when the reference pinhole is illuminated by the broadband source. It is defined as broadband PSF (BPSF). Finally, when a gray scale object illuminated by a broadband source transmits through a thin scattering medium, its speckle still shows the similar relationship as Eq. (3):

$$I_{mono} = O * BPSF \tag{5}$$

So the information of the object can be reconstructed by de-convolution of I_{mono} and BPSF. The conclusion is based on the assumption that Eq. (3) is satisfied for every wavelength that the broadband source contains. According to the Eq. (1), a thin scattering system can be considered as shift-invariant system only within the ME range. While ME range is variant with wavelength, the longer the wavelength is, the large the ME will be. For a paraxial object, Eq. (5) might be acceptable. While for an extended object, this might bring about more noise.

Secondly, for a color object and a color camera, the situation is similar. Usually, there

a built-in color filter in front of the CMOS sensor. It divides the light into red (R), green (G) and blue (B) channels. If the color object is also divided into RGB channels with the same characteristic as the built-in color filter, Eq. (5) is satisfied for each component. That is

$$I_i = O_i * BPSF_i \tag{6}$$

Here, *i* represents R, G or B. Thus, the BPSF and speckle pattern are detected for each channel. The original color object can be reconstructed by recombination of the de-convolution results for each channel.

4.2. The relation between the reconstructed quality and the bandwidth of illumination.

Broadband illumination will degrade the speckle and cause a lower contrast BPSF and lower SNR. Results with different bandwidth of illuminating light is compared and shown in Figure S3. The illuminating light source is generated by a white LED light passing through a 10 nm bandwidth filter @550nm (first column), a 60nm bandwidth filter @550nm (second column) and without filter (third column), respectively. Speckle patterns of a 100μm pinhole shown in first row are regarded as BPSFs. The speckle contrast (SC) decrease from 0.060 to 0.040 when bandwidth increases, which follows the relationship of $\mathrm{SC} \propto\sim \frac{1}{\sqrt{\delta\lambda}}$ [4]. For a speckle whose contrast is larger than 0.04, its grain distribution is still well recognized easily by human eyes.[5] The speckle patterns of object, shown in second row, have lower SC than BPSF because the object has higher sparsity than the pinhole. A lower contrast of raw speckle pattern leads to higher noise from CCD A/D conversion and worse reconstructed image, shown in third row. However, a common CCD with 8-bit dynamic range can resolve SC greater than 0.004 (when CCD is fully used of capability with the shot-noise ignored.). A CCD with higher dynamic range (12-bit) (Basler ACE 2040-90UM) is applied in the experiment to enhance the resolution of fine signal drift. The deconvolution process can efficiently extract signal from noise and retrieve the image.

4.3. The relation between the reconstructed FOV and the bandwidth of illumination.

To guarantee the successful of the de-convolution process, objects should locate within the ME range of the scattering medium. According to the Eq. (1), ME range is variant with wavelength, the longer the wavelength is, the large the ME will be. Supposed the system is illuminated by a broadband source ranging from λ_1 to λ_2 ($\lambda_1 < \lambda_2$). L_1 and L_2 is the ME range corresponding to these two wavelengths. An extended tested object of width L is located on the object plane and L is equal to L_2. It can get a perfect de-convolution reconstruction image under the source of wavelength λ_2. However, the part of object beyond the range of L_1 will be un-correlated when doing a de-convolution process with the wavelength of λ_2. And this part will turn out to be noise. To clarify this an additional experiment is demonstrated. The results are shown

in the 4th row of the Figure S3 for the reconstruction of an extended object illuminated by a white LED light passing through a 10 nm bandwidth filter @550nm (first column), a 60nm bandwidth filter @550nm (second column) and without filter (third column), respectively. It shows that a narrower band light source (with a filter (±10@532nm) would lead to a larger FOV and a higher SNR than the broadband (white LED light) source.

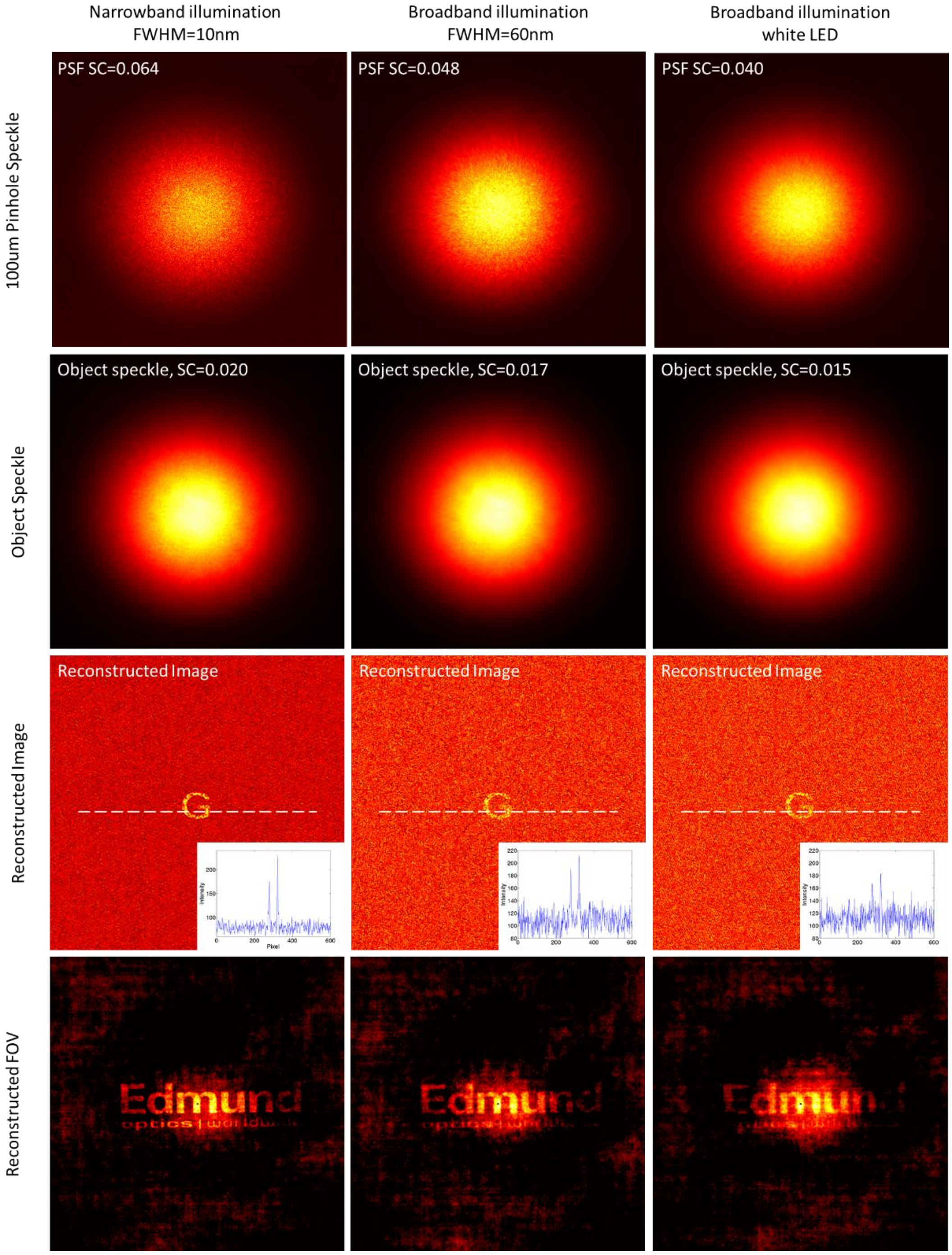

Figure S3. Results with different bandwidth of illuminating light. First column: PSF, object speckle and reconstruction result with a white LED light passing through a 10 nm bandwidth filter @550nm. Second column: PSF, object speckle and reconstruction result with white LED light passing through a 60nm bandwidth filter @550nm. Third column: PSF, object speckle and reconstruction result with

white LED light passing through without filter.

5. Methods to Optimize the SNR of the constructed image

As experimental suggestions, there are several methods to improve the quality of the reconstructed images by taking into account the ME range, contrast of raw speckle pattern and retrieval algorithms parameter. The following is the methods to optimize the SNR

(1) Our approach is based on an assumption that scatter medium is a linear shift-invariant system. A paraxial target with small size or a scatter with large ME range would satisfy to this assumption better.

(2) The speckle contrast (SC) relates to the bandwidth of the illumination source ($SC \propto \sim \frac{1}{\sqrt{\delta\lambda}}$)[4]. It also relates to the sparsity of the object ($SC \propto \sim \frac{1}{\sqrt{N}}$, N for sparsity)[3]. So a Narrow bandwidth light source (for example, a He-Ne laser passing through a rotating diffuser) is benefit for high quality imaging reconstruction. A low sparsity object could also increase the imaging quality, i.e., objects of less complexity and with smaller number of points. Anyway, if the dynamic range of the CCD is high enough and the readout noise is very low, one can ignore the influence of low contrast speckle pattern.

(3) Reducing the effective area of the diffuser with a diaphragm. The SC will increase with a larger grain of speckle pattern[3]. Besides, the objective lens in the proposed experimental setup is placed close enough to the diffuser. The small entrance pupil of the objective lens functionally limits the effective area of the diffuser.

(4) When using the Wiener filter for the deconvolution operation, property setting the value of k in Eq. (S2) will dramatically decrease the noise and improve the quality of the image.

6. FOV- Magnifying Lenses

In general, the CCD has a limited sensor area and can only capture part of the correlated speckles. If the speckle halo is partially captured, it will results in a smaller FOV compared with the theoretical limitation (ME range[1,6]). To enlarge the system's FOV, a magnifying lenses system is introduced. By fully captured of the speckle halo, the FOV angle can approach to the ME range.

The speckle loss leads to a smaller FOV. A simulation is presented to clarify the situation. Assuming that a point light source generates a Gaussian speckle pattern with waist of W_0 behind the diffuser. After it passes through the magnifying lenses, the waist of the speckle halo on the CCD is $W= W_0 \bullet M$. When the point source shifts away from axis, the speckle pattern on the CCD shifts accordingly and it may even exceed the receiving area of the CCD, as illustrated in Figure S4(a). Partly captured speckle pattern is less correlated compared with the fully captured speckle, leading to a reduced reconstruction. Simulation shows that a larger ratio of W to R (CCD size) would lead to a narrower FOV but a smaller W could enlarge the FOV, as illustrated in Figure S4 (b). The limits of a minimum W is to guarantee a fully resolved speckle

pattern that is received by the CCD. The speckle grain should be larger than two CCD pitches to meet Nyquist sampling criterion.

An experiment is performed as a further prove. By adjusting the distance between two lenses, the size of the speckle pattern W is controlled. For each speckle pattern a same FOV measurement process (the details can be referred to the manuscript) are performed and the results are shown in Figure S4(c). The data of ME curve in figure S4(c) is from our previous ME measurement. Curve of W=0.5R is the same as that of Figure 2 in the manuscript. Results in Figure S4(c) follow the trend of the simulation results. However, the experimental curves drop more rapidly as W gets larger. The reasons is that the spackle patterns captured in experiment are deviated from Gaussian profile. The experiment show further evidence to the previous conclusion: with the help of lenses system, when the speckle halo is controlled to a size of W=0.5R, the FOV can approach the ME range (here FOV=75mrad, ME=80mrad, FOV=0.93ME).

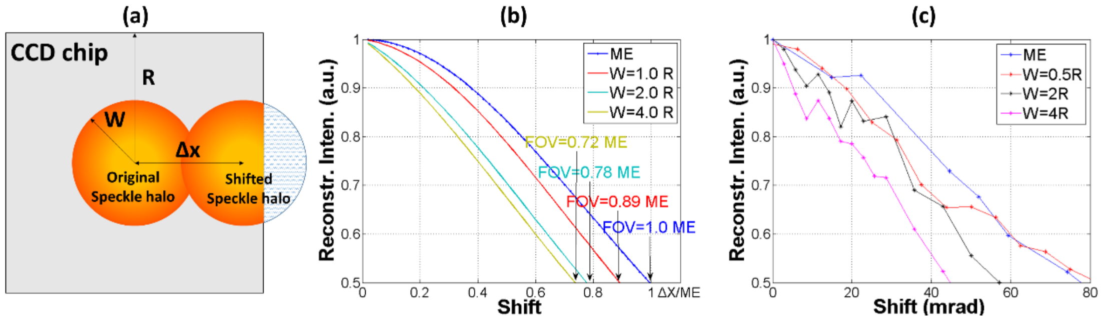

Figure S4. Relation between FOV and speckle halo size W. (a) Calculation model, illustration for the situation of the shifted speckle halo is exceed CCD area; (b) Simulation results for the relationship between FOV and W; (c) FOV measurement results for different speckle halo size W.

7. Video legends:

Supplementary Videos S1

A video illustrates the imaging construction result for a grayscale dynamic object. The object is a cartoon of a sparkle. It is generated by a SLM projector (Fig. 1c in the main text). The image is in-line reconstructed while the cartoon is playing on the projector. The synchronization between the reconstructed image and the object is well.

Supplementary Videos S2

A video illustrates the imaging construction result for color dynamic objects. The object is generated by a SLM projector (Fig. 1c in the main text). Objects consist of three circles. The colors of them are red, blue and green respectively. The three circles move in horizontal direction. When two of them overlap, the colors are mixed and colors of yellow (red + blue), cyan (green + blue) and magenta (red + blue) are generated. The reconstructed result is well consisted with the original objects, for both the shape and the colors.

References

1. Katz, O., Small, E., & Silberberg, Y., Looking around corners and through thin turbid layers in real time with scattered incoherent light. Nat. Photon., 6, 549-553

(2012).

2. Schott, S., Bertolotti, J., Léger, J. F., Bourdieu, L., & Gigan, S., Characterization of the angular memory effect of scattered light in biological tissues. Optics express, 23(10), 13505-13516. (2015).
3. Katz, O., Heidmann, P., Fink, M., & Gigan, S., Non-invasive single-shot imaging through scattering layers and around corners via speckle correlations. Nat. Photon., 8, 784-790 (2014).
4. J. W. Goodman, Speckle Phenomena in Optics: Theory and Applications (Roberts, 2007).Or Opt. Express, 3548, 10 February 2014, Vol. 22, No. 3
5. Wang, L., Tschudi, T., Halldorsson, T., & Petursson, P. R. Speckle reduction in laser projection systems by diffractive optical elements. Applied optics, 37(10), 1770-1775. (1998).
6. He, H. X., Guan, Y. F., & Zhou, J. Y., Image restoration through thin turbid layers by correlation with a known object. Opt. Express, 21, 12539-12545(2013).
